# A Novel, Portable MESH Nebulizer—An Alternative to Metered Dose Inhaler: Efficacy and Usability in Preschool Wheezers

**DOI:** 10.3389/fped.2020.598690

**Published:** 2020-12-10

**Authors:** Nicola Ullmann, Antonio Di Marco, Fabiana Columbu, Valentina Negro, Maria Beatrice Chiarini Testa, Valentina Panetta, Salvatore Tripodi, Ekaterina Potapova, Annalisa Allegorico, Paolo Maria Matricardi, Renato Cutrera

**Affiliations:** ^1^Pediatric Pulmonology & Respiratory Intermediate Care Unit, Sleep and Long Term Ventilation Unit, Academic Department of Pediatrics, Research Institute, Bambino Gesù Children Hospital, Rome, Italy; ^2^L'altrastatistica srl, Consultancy & Training, Biostatistics, Rome, Italy; ^3^Allergology Unit, Policlinico Casilino Hospital, Rome, Italy; ^4^Department of Pediatric Pneumology & Immunology, Charité—Universitätsmedizin Berlin, Berlin, Germany

**Keywords:** nebulizer, wheezing, childhood, metered dose inhaler (MDI), therapy

## Abstract

**Introduction and Objectives:** Wheezing episodes are the first causes of doctor's consultation in preschool age. Treatment is usually administered with a metered dose inhaler (MDI) spacer. At variance, many parents and doctors prefer to use a compressor nebulizer, which cannot be easily carried. The study is aimed at testing whether a pocket mesh nebulizer has similar efficacy and acceptability than a standard MDI device.

**Materials and Methods:** The IPAC study was a randomized, controlled, non-inferiority trial (number: 1616/2018, Ospedale Pediatrico Bambino Gesu'—IRCCS). The study had two arms: cases, using MicroAIR U100, and controls, using MDI+spacer device. Both devices were adopted for long-term treatment and for exacerbations. Follow-up was organized with clinical visits and a daily e-diary connected to an application for mobile phone.

**Results:** One hundred patients were enrolled. The frequency of asthmatic symptoms showed a non-inferiority for MicroAIR U100 group vs. MDI. Accordingly, no significant difference was found in the average % of days with cough, wheezing, breathlessness after exercise, days lost at school, and not-programmed visits. Considering only patients with >1 day with symptoms, no significant sdifferences were found in the number of exacerbations nor in the cumulative days with symptoms. The acceptance and usability of both devices have been favorable. However, the MDI+AeroChamber® device showed better acceptability.

**Conclusions:** Our study shows that MicroAIR U-100, a mesh nebulizer, has similar clinical efficacy but lower acceptance and usability than an MDI plus Aerochamber® in delivering therapy in preschool wheezers. Therefore, MicroAIR U-100 might be a valuable second choice, when the delivery of medication with an MDI plus Aerochamber® is not accepted, or wrongly used by the parents.

## Background

Viral infections of the upper and lower airways, along with wheezing, are the first causes of doctor's consultation in the preschool age (1). Their social and economic burden at worldwide level is enormous (2, 3). Preschool children with chronic disorders of the lower airways (e.g., asthma) often suffer from recurrent wheezing exacerbations (4, 5). Treatment for acute episodes is based on short-acting b2-agonists, usually administered, according to the international guidelines, with a metered dose inhaler (MDI) spacer (6–8). The same device must be used to daily deliver inhaled corticosteroids (ICS), i.e., to treat the underlying inflammatory chronic disorder (9, 10). At variance, many parents and some doctors choose to administer the ICS or b2-agonists through nebulization (11–14). The most frequent nebulizers used are based on a compressor, require electric power, take about 5–10 min for drug administration, and cannot be easily carried. Given these characteristics, nebulizers are normally used only at home, and their use is often limited.

The study is aimed at testing whether a nebulizer that can be easily carried everywhere has a similar or higher efficacy and acceptability than a standard MDI device. A pocket mesh nebulizer (MicroAIR U100) has been recently developed. MicroAIR U100 produces an aerosol with MMAD of 4.5 μm and takes about 5 min to deliver the treatment (e.g., ICS or salbutamol). The efficiency and acceptability of MicroAIR U100 in a real-life setting are, however, unknown but essential in order to establish whether it can be a good alternative when treating children with recurrent wheezing.

Therefore, the “Inhalation Devices in Preschool Asthmatic Children (IPAC)” study was designed to investigate if daily therapy, delivered through a MicroAIR U100 Mesh nebulizer, is higher or not inferior than MDI, in the control of wheezing and in reducing the episode's severity in preschool children. Finally, we explored the non-inferiority in terms of device's usability, acceptability, and tolerability.

## Methods

### Study Design and Population

**Figure 1:**
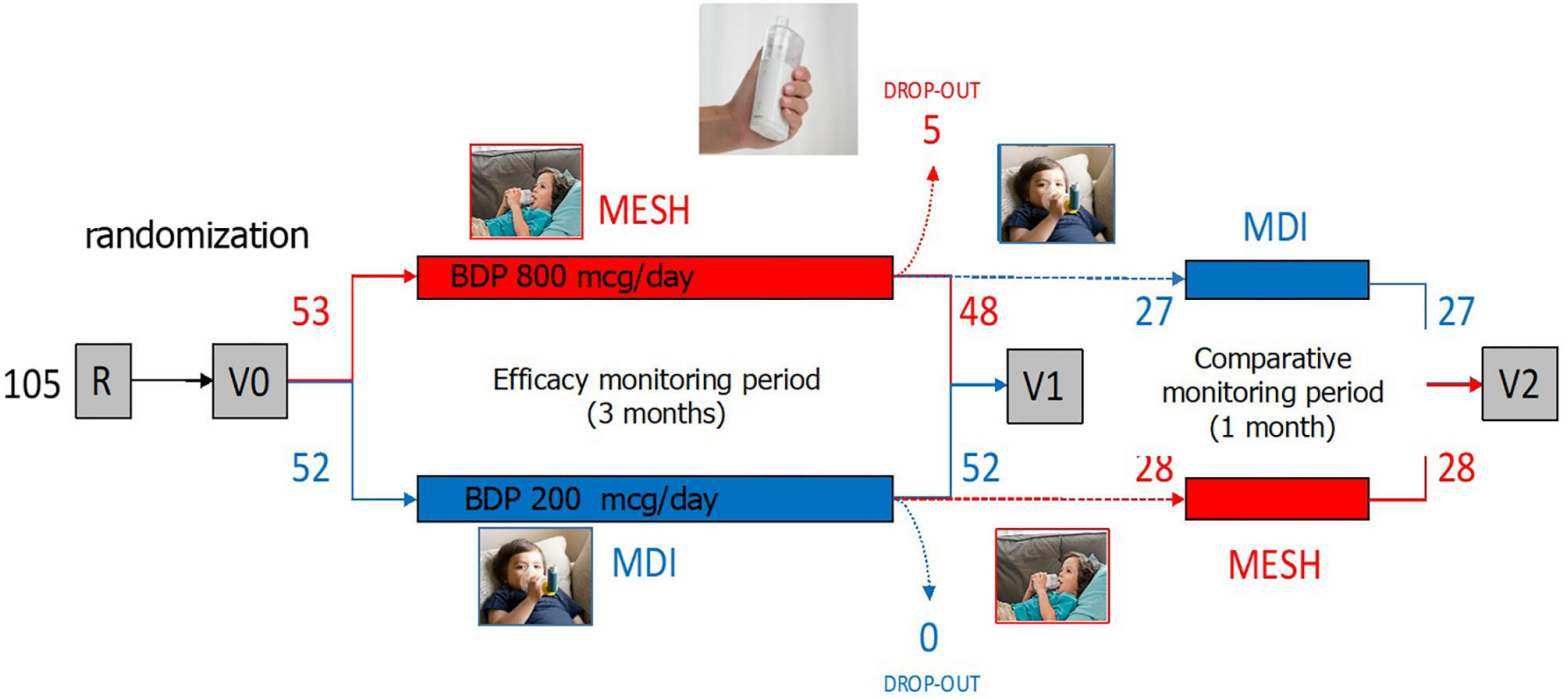
The IPAC study has been a randomized, controlled, non-inferiority, investigator-initiated trial (RCT, IIT). The study had two arms: (A: red) patients using MicroAIR U100 (OMRON®) (hereafter labeled as the “MicroAIR” group), and (B: blue) patients using the metered dose inhaler (MDI)+spacer device (Aerochamber®–L'Espace, Air Liquide Healthcare) (hereafter labeled as the “MDI” group). The study is structured in a recruitment and time “0” visits (R0 and V0), an efficacy monitoring period (EMP), a post EMP visit at time “1” (V1) visit, a comparative monitoring period (CMP), a post CMP visit at time “2” (V2) visit (image taken from ©Shutterstock, September 2020).

The IPAC study has been a randomized, controlled, non-inferiority, investigator-initiated trial (RCT, IIT) conducted in the Department of Pediatric Pulmonology in the “Ospedale Pediatrico Bambino Gesù” in Rome, Italy, between November 2018 and July 2019. The study had two arms: cases, using MicroAIR U100 (OMRON®), and controls, using MDI+spacer device (AeroChamber® Plus, Trudell Medical). The study is structured in distinct periods: a recruitment and time “0” visits (R0 and V0), an efficacy monitoring period (EMP), a post EMP visit at time “1” (V1) visit, a comparative monitoring period (CMP), a post CMP visit at time “2” (V2) visit (Figure 1). The inclusion criteria were (1) a history or recurrent wheezing (>3 reported episodes in the last 12 months), (2) age between 25 and 72 months, (3) sufficient comprehension of the Italian language, (4) availability of a smart-phone, and (5) consensus to participation. The exclusion criteria were (1) anatomic malformation causing a chronic bronchial obstruction, (2) severe chronic diseases (i.e., cancer, primary immunodeficiency), (3) contraindication for the use of beta sympathomimetic drugs, and (4) intention to move away from Rome during the monitoring period.

### Questionnaires and e-Diary

A specifically dedicated web-platform (U100study-CARD) (ELMACOM S.r.l., Guidonia, Italy) has been produced and used for the questionnaires at V0, V1, and V2. These questionnaires focused on the respiratory symptoms and their impact on the family's quality of life (V0, V1,) and on the acceptability and usability of the MicroAIR U100 or the MDI-AeroChamber® device (child's tolerance, parents' satisfaction, etc.). During the monitoring periods, parents have filled every evening an e-Diary “RespiMonitor” (ELMACOM S.r.l., Guidonia, Italy) in a specific App with their child's symptoms, medication use, and other parameters. Hospitalization and doctor's appointments have also been registered. Compliance to compilation has been supported by regular alerts, and patients with a low compilation rate have also been contacted by the study nurse, in the attempt to improve compliance. Patients have been considered as drop-out if they did not participate at V1 visit.

### Treatment

ICS (control therapy) and salbutamol as needed (rescue medication), prescribed according to international guidelines, have been administered with MicroAIR U100-Mesh nebulizer or a standard MDI (beclomethasone dipropionate) + spacer device (AeroChamber® Plus), according to the patient's allocation. Oral corticosteroids have been also prescribed as needed. The OMRON®'s “MicroAIR U100” nebulizer is a CE certified and thoroughly tested device for the standard use in infants and adults (Supplementary Figure 1). During the visit, a study nurse has trained the parents to properly set-up, use, and clean both devices.

### Primary and Secondary Outcomes

The primary outcome of the study was the proportion of days with asthma-like symptoms during the EMP. A long list of secondary outcomes has been examined, including the use of salbutamol or other rescue medication, the severity of the respiratory symptoms, and the impact of the symptoms on the family's quality of life. The usability and tolerability of the MicroAIR nebulizer has been also compared to that of the MDI between the two groups at EMP/V1 and within each study group during/at CMP/V2.

### Ethics

The study has been run according to the Helsinki Declaration, the rules of Good Clinical Practice, and the CONSORT guidelines. The study has been approved by the Ethical Commission of OPBG, (number: 1616/2018).

### Statistical Methods

Sample size has been computed on primary outcome. The mean expected frequency of days (on 90 days) with asthma-like symptoms is 31.11% with an sd of 8.89%. Considering a non-inferiority limit of 5 days (5.56%), a two-sided 95% CI and a power of 80%, the estimated required minimum sample size was 41 children per group, with a 20% drop out, was planned to recruit 104 children. Only children who suffered from asthma symptoms during the EMP have been admitted to the CMP and V2 visit.

Data were summarized as numbers (n) and frequencies (%) if they were categorical and as mean/median and standard deviation (SD)/interquartile range (IQR) if quantitative. Chi-squared test or Fisher test were used to evaluate the association of categorical data between groups. Mann Whitney U-test or *T*-test were used to compare quantitative variables. The primary endpoint is the non-inferiority of mesh nebulizer vs. MDI in frequency of days free of symptoms. The percentage days with symptoms, reported in the e-diary, was calculated as number of days with symptoms on all observed period ^*^ 100. The mean difference between the two groups and the relative 95% CI was reported. Non-inferiority is assessed if the upper limit of 95% CI of the difference between the mesh nebulizer and MDI does not exceed 5.56%. As for sensitivity analysis, a non-parametric evaluation was provided basing on the Hodges–Lehmann estimator and Moses'. Frequencies are ever calculated on total number registered. Multilevel mixed effects were applied to take into account the repeated measures of the same patients for analysis on an episode's duration. The answers to questions on acceptance and usability of the devices have been dichotomized, considering the highest class (most positive) of response apart from the remaining ones. Analysis was made on full analysis set (FAS) and per protocol (PP) population. To evaluate the effect of variables not balanced by randomization, a fractional regression was implemented considering groups and the three variables significant in Table 1 (emergency respiratory event, missed school days, and diagnosed pseudocroup) as independent factors. Results of the multivariate analysis for the primary outcome are shown in Table 2. A *p* < 0.05 was considered statistically significant. Statistical analyses were performed with SAS 9.4 software.

**Table 1:** Characteristics of the patient population in the last 12 months.

		**MicroAir U100**	**MDI**	***P*-value**
		***n* = 53**	***n* = 52**	
Male gender	%, (n)	64.2 (34)	46.2 (24)	0.064
Age	Mean ± SD	3.4 ± 1.0	3.3 ± 1.2	0.690
Cough outside cold/infection	%, (n)	84.9 (45)	78.8 (41)	0.420
Cough after waking up	%, (n)	83.0 (44)	88.5 (46)	0.426
Wheezing	%, (n)	100.0 (53)	100.0 (52)	
3–6	%, (n)	90.6 (48)	80.8 (42)	0.264
7–12	%, (n)	5.7 (3)	15.4 (8)	
>12	%, (n)	3.8 (2)	3.8 (2)	
**WHEEZING-INDUCED TROUBLE SLEEPING**				
Never	%, (n)	28.3 (15)	23.1 (12)	0.527
<1 night/week	%, (n)	58.5 (31)	55.8 (29)	
1 or > nights/week	%, (n)	13.2 (7)	21.2 (11)	
Wheezing-induced trouble speaking	%, (n)	24.5 (13)	40.4 (21)	0.083
Exercise-induced wheezing	%, (n)	26.4 (14)	38.5 (20)	0.187
Shortness of breath	%, (n)	100.0 (53)	100.0 (52)	-
**DIAGNOSED ASTHMA**				
One	%, (n)	17.0 (9)	13.5 (7)	0.207
Many	%, (n)	7.5 (4)	19.2 (10)	
**DIAGNOSED ASTHMA BRONCHITIS**				
One	%, (n)	24.5 (13)	13.5 (7)	0.149
Many	%, (n)	75.5 (40)	86.5 (45)	
Diagnosed pseudocroup				
Never	%, (n)	94.3 (50)	94.2 (49)	0.050
One	%, (n)	5.7 (3)	-	
Many	%, (n)	-	5.8 (3)	
**FREQUENCY RESPIRATORY SYMPTOMS LAST 12 MONTHS**				
Never	%, (n)	-	1.9 (1)	0.37
3–6	%, (n)	92.5 (49)	80.8 (42)	
7–12	%, (n)	5.7 (3)	11.5 (6)	
>12	%, (n)	1.9 (1)	5.8 (3)	
Doctors' visit due to respiratory symptoms	%, (n)	100.0 (53)	100.0 (52)	0.317
**FREQUENCY DUE TO ASTHMA AND WHEEZING**				
1–2	%, (n)	9.4 (5)	11.5 (6)	0.520
3–6	%, (n)	86.8 (46)	80.8 (42)	
7–12	%, (n)	3.8 (2)	3.8 (2)	
>12	%, (n)	-	3.8 (2)	
**EMERGENCY RESPIRATORY EVENTS**				
Never	%, (n)	64.2 (34)	36.5 (19)	0.039
1–2	%, (n)	24.5 (13)	40.4 (21)	
3–6	%, (n)	9.4 (5)	21.2 (11)	
7–12	%, (n)	1.9 (1)	1.9 (1)	
**HOSPITALIZATION DUE TO RESPIRATORY SYMPTOMS**				
Never	%, (n)	83.0 (44)	73.1 (38)	0.456
1–2	%, (n)	11.3 (6)	19.2 (10)	
3–6	%, (n)	5.7 (3)	7.7 (4)	
Respiratory symptom medication	%, (n)	100.0 (53)	100.0 (52)	0.456
**MISSED SCHOOL DAYS**				
0 days	%, (n)	15.1 (8)	1.9 (1)	0.008
1–5 days	%, (n)	22.6 (12)	7.7 (4)	
6–10 days	%, (n)	7.5 (4)	9.6 (5)	
>10 days	%, (n)	54.7 (29)	80.8 (42)	
Sneezing outside cold	%, (n)	64.2 (34)	63.5 (33)	0.941
Eye symptoms	%, (n)	32.1 (17)	30.8 (16)	0.885
**DAILY LIFE DISRUPTION DUE TO NASAL SYMPTOMS**				
Not at all	%, (n)	47.2 (25)	48.1 (25)	0.100
A little bit	%, (n)	37.7 (20)	21.2 (11)	
Enough	%, (n)	15.1 (8)	26.9 (14)	
A lot	%, (n)	-	3.8 (2)	
Nasal with no eye symptoms	%, (n)	50.9 (27)	53.8 (28)	0.766

**Table 2:** Results of multivariate analysis for the primary outcome.

**Days with symptoms**	Estimated mean diff (95% CI)^*^
Model 1	−0.81 (−6.29; 4.66)
Model 2	−0.74 (−6.86; 5.39)

* Difference between the two group estimate by fractional regression on PP population.

Model 1 adjust for emergency respiratory events. Missed school days diagnosed pseudocrop.

Model 2 adjust for emergency respiratory events.

## Results

### Characteristics of the Study Population

One hundred five patients with recurrent wheezing met the inclusion criteria and were 1:1 randomly assigned to the case (*n* = 53) or the control (*n* = 52) group, receiving the Micro AIR U-100 MESH nebulizer or the MDI+AeroChamber®, respectively. Five patients among the Micro AIR U-100 MESH group and zero among the MDI group were considered as drop-out. Out of the 100 patients participating at V1 visit, 55 were invited to continue the study and completed the CMP and the V2 (Figure 1). No significant differences were observed among the Micro AIR U100 group and the MDI group with regard to age and gender and most markers of disease severity. However, the AIR U100 group was less frequently affected by pseudocroup and exacerbations requiring emergency room visit or loss of days at school or kindergarten (Table 1).

### Adherence to Monitoring

**Figure 2:**
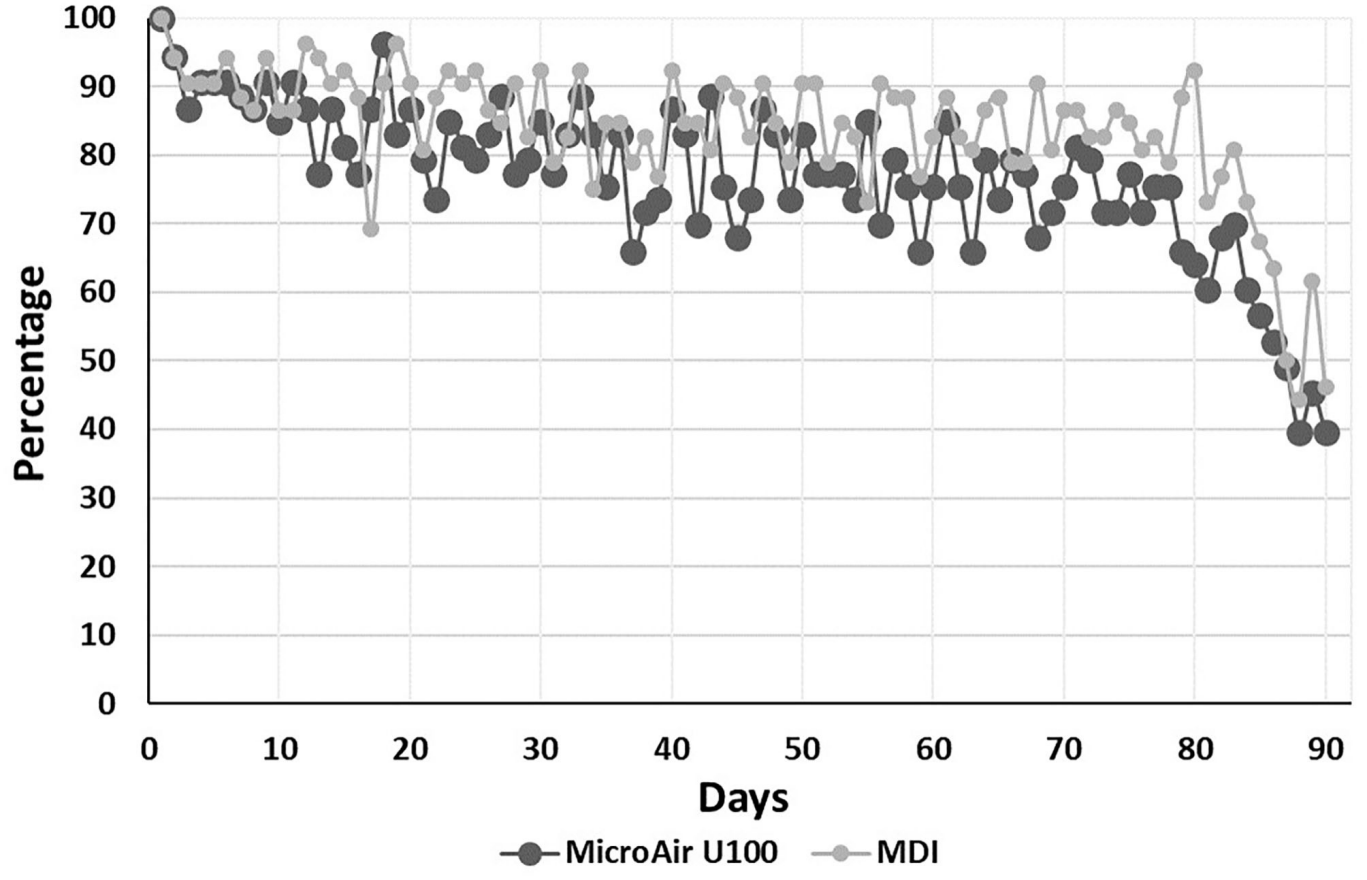
Adherence to e-Diary compilation during the 90-day-long EMP.

The median (IQR) number of days monitored during the EMP was 75 (IQR 62–82) and 76.5 (IQR 71–83) (*p* = 0.164) in the MicroAir U 100 and MDI patients, respectively. The average adherence to e-Diary compilation was kept above 70%, starting with about 90% in the first 10 days and declining to about 75% at the 80th day of compilation (Figure 2).

### Response to Therapy (Asthma Control)

**Figure 3:**
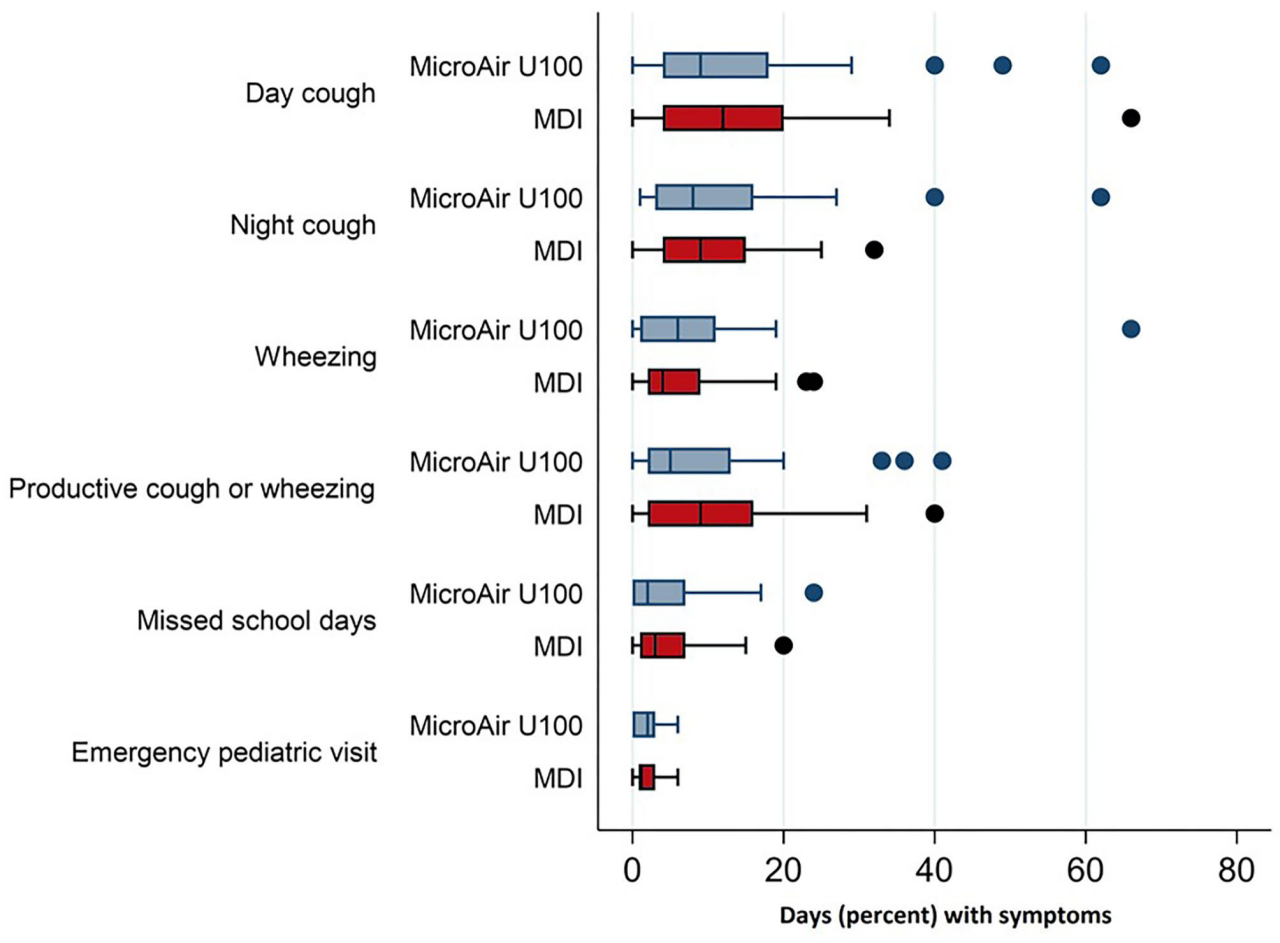
Frequency of asthmatic symptoms and asthma-related events in children with recurrent wheezing treated with microAIR U100 or MDI.

During the whole EMP (90 days), the frequency of days with asthmatic symptoms showed a non-inferiority for the MicroAIR U100 group vs. MDI. This outcome was confirmed also by Hodges–Lehmann estimator and did not change when the actual number of recorded days was used as a basis for calculations (MicroAIR U100 = 13.2 ± 18.9%; MDI = 15.9 ± 18.2%) (Table 3). Accordingly, no significant difference was found in the average percentage and cumulative days with diurnal or nocturnal cough, wheezing, breathlessness after exercise, days lost at school, not-programmed visits (Figure 3), and with the whole set of parameters examined as secondary outcomes (Supplementary Table 1). Considering only patients with >1 day with symptoms in the PP population, no significant difference was found in the number of events in the MicroAIR vs. the MDI group (3.0 ± 1.6; 3.7 ± 2.4; p = 0.120). Similarly, no significant difference was found in the duration of an exacerbation (5.62 ± 1.09; 4.14 ± 0.95; diff −1.48 CI 95% −4.3; 1.4 *p* = 0.306). These outcomes were also confirmed by analyses when expanded to the whole FAS population or when they were limited to the subset of exacerbation characterized by wheezing symptoms.

**Table 3:** Primary outcome [full analysis set (FAS) and per protocol (PP) population]; frequency of days with symptoms on the all observational period (90 days) comparing the micro AIR group vs. the MDI group.

**Days with symptoms**	**Summary**	**MicroAir U100**	**MDI**	Mean diff (95% CI)^*^	**Hodges–Lehmann estimator (95% CI)**
	**statistics**	***n* = 53**	***n* = 52**		
FAS−90 days (%)	Mean ± SD	10.6 ± 15.8	12.6 ± 14.4	−1.96 (−7.81; 3.89)	−1.11 (−5.56; 0.00)
		***n*** **=** **48**	***n*** **=** **52**		
PP−90 days (%)	Mean ± SD	11.6 ± 16.3	12.6 ± 14.4	−0.96 (−7.05; 5.12)	1.11 (−2.22; 4.44)

* *The upper limit of CI to define the non-inferiority was set at 5.56; in no analysis has it been reached*.

### Devices' Acceptance and Usability

**Figure 4:**
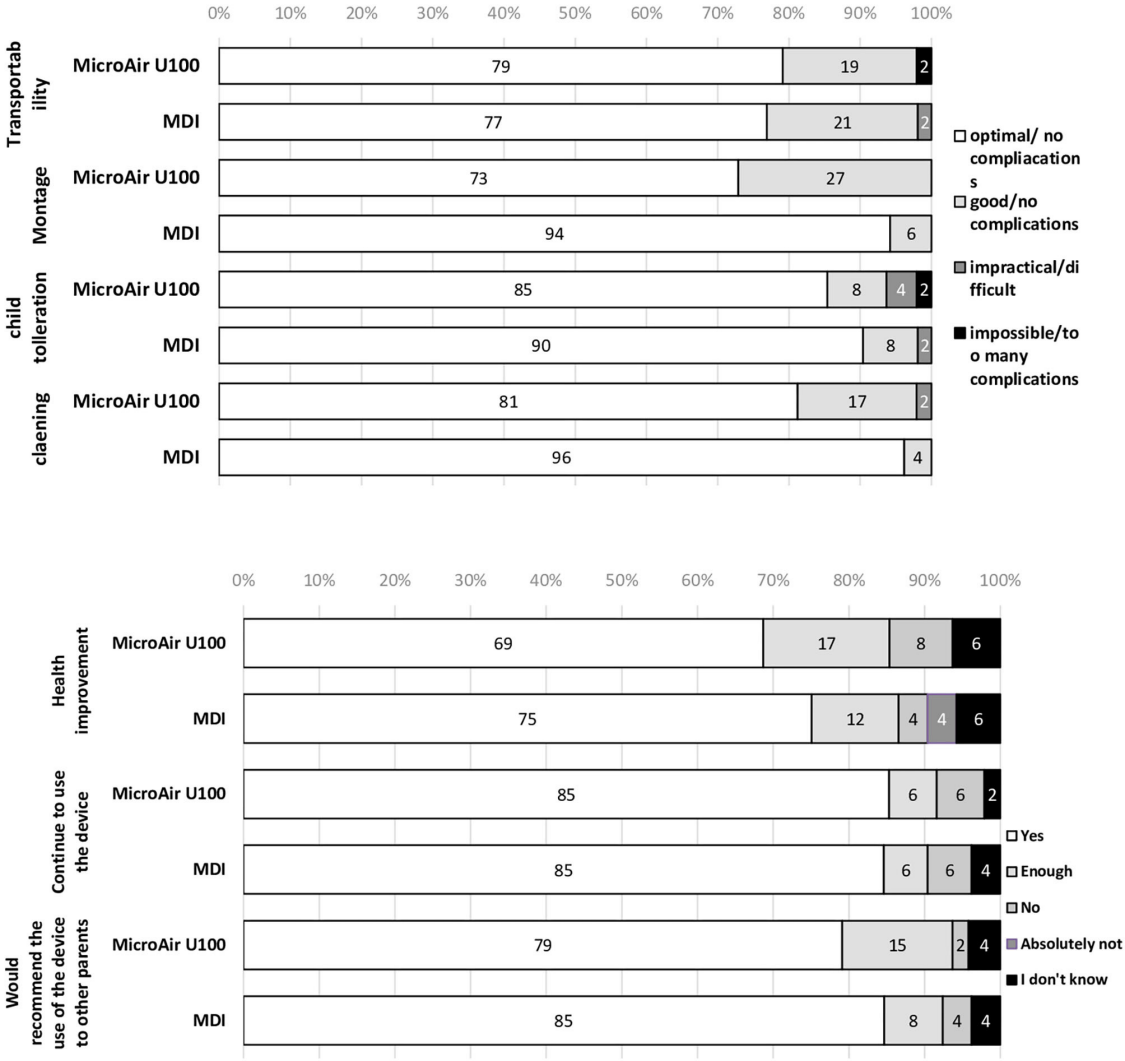
Acceptance and usability of microAIR U100 or MDI devices in delivering drug therapy in children with recurrent wheezing during a 90-day-long EMP.

The acceptance and usability of both devices have been favorable, considering that only one patient in the MicroAir group reported major problems in montage, daily use, and cleaning of the nebulization device at V1 (Figure 4). However, over 95% of the MicroAir group, but only about 75% of the MDI group, considered the assembling of the device as absolutely easy (Supplementary Table 2). Among the 55 families participating in the CMP and the V2, acceptability and usability of the MDI+AeroChamber® device were higher than those of the MicroAIR U-100 nebulizer (Supplementary Table 3).

## Discussion

### Major Findings

We undertook a case-control, cross-over study of a mesh nebulizer (MicroAIR U-100) in 105 preschool Italian children with recurrent wheezing, monitored with an e-Diary, during 3 months to test therapy efficacy, and an additional month to test acceptance and usability in comparison with a metered dose inhaler device. The patients' adherence to the compilation of the e-Diary was excellent. We observed no inferior efficacy of the MESH nebulizer compared to the MDI in controlling respiratory disease. The usability and the child's acceptance of the MESH nebulizer was good but slightly inferior than those on MDI.

### Efficacy

The frequency of days with wheezing was clearly not inferior in cases (MESH) than in controls (MDI). This primary outcome was further reinforced by the observation that none of the secondary outcomes showed a difference in the efficacy of the MESH nebulizer, when compared to the MDI. Moreover, no difference was found in the length of exacerbations. Our results suggest that not only the frequency, but also the severity of the wheezing episodes was similar, independently from the type of devices they used.

### Acceptance

More than 75% of the cases accepted very well the daily use of their mesh nebulizer during the 90 days of the EMP. However, acceptance rate was significantly higher among the controls. This outcome was confirmed by the intra-patient analysis done after the CMP in children with more severe symptoms. This outcome is not surprising, considering the much longer time (5 min) required by the MESH nebulizer to deliver treatment, and it is in line with previous literature (12–15). Moreover, the MESH nebulizer was used not only to deliver rescue medication, but every single day, to administer controller therapy. Possibly, for continuous treatment, families could be more compliant to a daily treatment with MDI + AeroChamber®. Given this premise, the good acceptance of the MESH MicroAIR U-100 nebulizer may be well explained by its portability, the absence of noise during its use, and the possibility of delivering drug in any child position. According to the side effects, no adverse effects were described by parents or caregivers. However, Castro-Rodriguez JA et al. reported a significant heart rate increase associated with the use of nebulizers (16).

### Usability

Over 75% of the parents perceived the assembly and use of the mesh MicroAIR U-100 nebulizer as easy and user friendly. However, the MDI was significantly superior with regard to the cleaning procedures. This outcome was confirmed by the intra-patient analysis. Again, this result is not surprising, considering that the MDI and its AeroChamber® requires only fast cleaning, while the nebulizer requires removal of the filter and a careful cleaning. The high viscosity of the Beclomethasone preparation for nebulization may explain this outcome. Other aspects, like the use of a battery to be recharged and the complexity of the assembly, may have contributed to the lower usability of the mesh nebulizer. On the other side, the portability of the mesh nebulizer has been well judged by the parents, who considered it as good as the MDI plus AeroChamber®. This characteristic of the mesh MicroAIR U-100 nebulizer is unique if compared to the compressor nebulizers and has been thoroughly analyzed elsewhere (17). According to the previous considerations, it should be highlighted that parents need to be well-trained on the correct use of the MicroAIR U-100 nebulizer to avoid malfunction.

### Positioning MicroAIR U-100

The results of our study guide further considerations in the management of preschool wheezers. The superior usability and acceptance of the MDI, confirmed in our study, further reinforce the priority given to the MDI plus AeroChamber® by the international guidelines in the treatment of wheezing in preschool children. However, the excellent efficacy and the good usability and acceptance of the MicroAIR U-100 prompt us to consider its use as a valuable alternative to basal and acute treatment when the MDI plus AeroChamber® is either not accepted or wrongly used. In fact, in real life, especially young children are often too active to stay calm and still. Therefore, a system that guarantees drug delivery for a longer period, such as 5 min, in some patients, could be more effective. Whether the nebulized treatment should be limited to rescue medication only or to both controller and rescue medication needs to be examined in further studies. However, possibly some patients might find more personal benefit from nebulized treatment, especially for acute treatment.

### Strengths and Limitations

We must acknowledge some limitations of our study design. First, the use of the mesh Micro-AIR nebulizer was prescribed also for controller therapy. Therefore, we cannot say whether the observed difference in usability and acceptance of the MESH vs. MDI device would have disappeared in the case of shorter, occasional use of the devices themselves. Moreover, we did not have the possibility to check if families switched to one or other treatment during exacerbations. Second, participation in the study was accompanied by a thorough training of the parents and an alerting system encouraging adherence to the study itself. The generalizability of our conclusion to real-life setting should be examined in observational studies performed under real-life conditions. Third, the frequency of wheezing days observed in our study population was about half of the one predicted. This “low” level of symptoms may be partially explained by the better disease control achieved through the participation itself to the study and by the fact that the 2018–2019 winter and spring seasons have been exceptionally mild in Rome, thus reducing one of the most important risk factors of respiratory symptoms. Last, given the study design, we could investigate the short-term, but not the long-term, durability of the nebulizer, and we could not perform a cost-benefit analysis.

## Conclusions

Our study shows that MicroAIR U-100, a mesh nebulizer, has similar clinical efficacy but lower acceptance and usability than an MDI plus Aerochamber® in delivering controller and rescue therapy over several months in children with recurrent wheezing. Therefore, MicroAIR U-100 might be a valuable second choice in the treatment of preschool children with recurrent wheezing, when the delivery of medication with an MDI plus Aerochamber® is not accepted by young and not compliant children, or wrongly used by the parents.

## Data Availability Statement

The datasets presented in this article are not readily available because Datasets not to be shared with third parties outside if the study. Requests to access the datasets should be directed to Nicola Ullmann, nicola.ullmann@opbg.net.

## Ethics Statement

The studies involving human participants were reviewed and approved by Ethical Commission of Bambino Gesù Children Hospital. Written informed consent to participate in this study was provided by the participants' legal guardian/next of kin.

## Author Contributions

RC and PM conceived and designed the study. PM wrote the first draft of manuscript with input from all the co-authors. NU, AD, VN, and MC enrolled patients, conducted the study, and collected data. NU contributed to the writing of the manuscript. ST worked out the informatics platform and all the IT related aspects. FC was the study nurse of the study and collected data. VP performed data management and statistical analysis of the study. All authors read and approved the final manuscript.

## Conflict of Interest

RC and PM reports grants and personal fees from OMRON Healthcare. ST reports personal fee by Elmacom S.r.l. The remaining authors declare that the research was conducted in the absence of any commercial or financial relationships that could be construed as a potential conflict of interest.
