# Supplementary material for: A Novel, Portable MESH Nebulizer—An Alternative to Metered Dose Inhaler: Efficacy and Usability in Preschool Wheezers
Source: Front Pediatr. 2020 Dec 10;8:598690. doi: 10.3389/fped.2020.598690 (PMC7758231; doi:10.3389/fped.2020.598690)

Supplementary Material

Table S1 - Number of days with symptoms within registered days.

	**Summary Statistics**	**MicroAir U100**	**MDI**	**P value**
		n=48	n=52	
**Day cough**	N	35 (72.9%)	42 (80.8%)	
	Median (IQR)	11.9 (6-24)	14.5 (6-28)	0.856
**Night cough**	N	35 (72.9%)	42 (80.8%)	
	Median (IQR)	9.9 (5-19)	9.9 (4-24)	0.717
**Wheezing**	N	35 (72.9%)	42 (80.8%)	
	Median (IQR)	6.5 (2-13)	6.1 (2-11)	0.61
**Productive cough or wheezing**	N	35 (72.9%)	42 (80.8%)	
	Median (IQR)	6.3 (2-18)	8.2 (2-20)	0.685
**No symptoms**	N	35 (72.9%)	42 (80.8%)	
	Median (IQR)	11.3 (5-19)	14.0 (4-24)	0.801
**Missed school days**	N	35 (72.9%)	42 (80.8%)	
	Median (IQR)	4.3 (0-10)	3.6 (1-8)	0.463
**Emergency pediatric visit**	N	35 (72.9%)	42 (80.8%)	
	Median (IQR)	2.0 (0-4)	1.6 (0-4)	0.96
**Emergency PS visit**	N	35 (72.9%)	42 (80.8%)	
	Median (IQR)	0.0 (0-0)	0.0 (0-0)	0.438
**Clenil**	Median (IQR)	98.1 (92-99)	98.7 (93-100)	0.729
**Montelukast**	Median (IQR)	17.8 (0-95)	6.0 (0-96)	0.754

**Required Broncovaleas/Ventolin**	Median (IQR)	10.6 (4-18)	8.6 (0-16)	0.943
**Required Bentelan o Sintredius**	Median (IQR)	0.0 (0-4)	0.0 (0-3)	0.489
**Required other Asthma care**	Median (IQR)	0.0 (0-5)	2.3 (0-8)	0.069

Table S2 – Acceptance and Usability of MicroAir and MDI devices, examined at V1.

	Summary statistics	MicroAir U100 n=48	MDI n=52	P value
**Trasportabilità del dispositive**				
Ottimale/priva di complicazioni	% (n)	79.2% (38)	76.9% (40)	0,553
Buona/priva di complicazioni/priva di complicazioni	% (n)	18.8% (9)	21.2% (11)	
Poco pratica/difficile	% (n)	0.0% (0)	1.9% (1)	
Impossibile/troppe complicazioni	% (n)	2.1% (1)	0.0% (0)	
**Il montaggio delle componenti del dispositive, secondo Lei, è stata**				
Priva di problemi	% (n)	72.9% (35)	94.2% (49)	0,004
Lievi problemi immediatamente risolti	% (n)	27.1% (13)	5.8% (3)	
Impossibile	% (n)	0.0% (0)	0.0% (0)	
**Che tipo di problemi ha avuto nel montaggio del dispositive?**				
Nessun problema	% (n)	79.2% (38)	98.1% (51)	0,053
Problemi nel montaggio dell'apparecchio	% (n)	12.5% (6)	1.9% (1)	
Problemi nell'accensione dell'apparecchio	% (n)	4.2% (2)	0.0% (0)	
Problemi nel posizionamento corretto del/la bambino/a	% (n)	2.1% (1)	0.0% (0)	
Problemi nel posizionamento corretto dell'apparecchio	% (n)	2.1% (1)	0.0% (0)	
**Come ha tollerato il suo/a bambino/a l'impiego del dispositive**				
Tollerato bene senza alcun problema	% (n)	85.4% (41)	90.4% (47)	0,663
Dopo alcune iniziali difficoltà lo ha tollerato bene senza ulteriori problemi	% (n)	8.3% (4)	7.7% (4)	
Ha pianto o si è agitato inizialmente, ma si è potuto completare la nebulizzazione	% (n)	4.2% (2)	1.9% (1)	
Non ha tollerato l'apparecchio e la nebulizzazione è stata impossibile	% (n)	2.1% (1)	0.0% (0)	
**La pulizia del dispositive e delle sue componenti è stata:**				
Priva di problemi	% (n)	81.3% (39)	96.2% (50)	0,055
Lievi problemi immediatamente risolti	% (n)	16.7% (8)	3.8% (2)	
Alquanto difficile	% (n)	2.1% (1)	0.0% (0)	
Impossibile	% (n)	0.0% (0)	0.0% (0)	
**Quando il/la Suo/a bambino/a ha inalato il Ventolin con il dispositive, i suoi sintomi respiratori sono migliorati?**				
Sì: ha potuto respirare liberamente dopo la nebulizzazione	% (n)	45.8% (22)	61.5% (32)	0,045
Parzialmente ha potuto respirare meglio dopo la nebulizzazione	% (n)	10.4% (5)	19.2% (10)	
No: non ho potuto osservare differenze tra prima e dopo la nebulizzazione	% (n)	10.4% (5)	7.7% (4)	
Non saprei	% (n)	33.3% (16)	11.5% (6)	
**L'uso del dispositive ha permesso al/la Suo/a Bambino/a di dormire meglio?**				
Si	% (n)	54.2% (26)	53.8% (28)	0,767
Abbastanza	% (n)	29.2% (14)	30.8% (16)	
Direi di no	% (n)	4.2% (2)	7.7% (4)	
Assolutamente no	% (n)	2.1% (1)	0.0% (0)	
Non saprei, il/la mio/a bambino/a non ha avuto disturbi del sonno	% (n)	10.4% (5)	7.7% (4)	
**L'uso del dispositive ha migiorato il benessere del/la Suo/a Bambino/a?**				
Si	% (n)	52.1% (25)	61.5% (32)	0,303
Abbastanza	% (n)	37.5% (18)	23.1% (12)	
Direi di no	% (n)	8.3% (4)	5.8% (3)	
Assolutamente no	% (n)	-	1.9% (1)	
Non saprei	% (n)	2.1% (1)	7.7% (4)	
**Ritiene che l'uso del dispositive sia stato utile per il/la Suo/a Bambino/a?**				
Si	% (n)	68.8% (33)	75.0% (39)	0,509
Abbastanza	% (n)	16.7% (8)	11.5% (6)	
Direi di no	% (n)	8.3% (4)	3.8% (2)	
Assolutamente no	% (n)	-	3.8% (2)	
Non saprei	% (n)	6.3% (3)	5.8% (3)	
**Vorrebbe usare ancora in futuro il dispositive per il/la Suo/a Bambino/a?**				
Si	% (n)	85.4% (41)	84.6% (44)	0,964
Abbastanza	% (n)	6.3% (3)	5.8% (3)	
Direi di no	% (n)	6.3% (3)	5.8% (3)	
Assolutamente no	% (n)	-	-	
Non saprei	% (n)	2.1% (1)	3.8% (2)	
**Prima di partecipare a questo studio, aveva utilizzato altri nebulizzatori?**				
No	% (n)	16.7% (8)	38.5% (20)	0,015
Si	% (n)	83.3% (40)	61.5% (32)	
**Se SI', ritiene che la praticità e l'efficacia del dispositive sia stato, in confronto con l'impego di altri nebulizzatori:**				
Migliore	% (n)	80.0%(32/40)	68.8%(22/32)	0,385
Leggermente migliore	% (n)	10.0%(4/40)	9.4%(3/32)	
Simile	% (n)	5.0%(2/40)	18.8%(6/32)	
Leggermente peggiore	% (n)	2.5%(1/40)	-	
Peggiore	% (n)	2.5%(1/40)	3.1%(1/32)	
**Consiglierebbe l'impiego dell' dispositivel ad altri genitori di bambini con problemi respiratori simili a quelli del/la Suo/a bambino/a?**				
Si	% (n)	79.2% (38)	84.6% (44)	0,698
Probabilmente si	% (n)	14.6% (7)	7.7% (4)	
Probabilmente no	% (n)	2.1% (1)	3.8% (2)	
Assolutamente no	% (n)	-	-	
Non saprei	% (n)	4.2% (2)	3.8% (2)	

Table S3 – Acceptance and Usability of the MicroAIR MDI devices, within-patient comparison (V2 vs V1).

	Summary statistics	MicroAIR U100<br>n=55	MDI<br>n=55	P value
**Trasportabilità del dispositive**				
Ottimale/priva di complicazioni	%, (n)	74.5% (41)	81.8% (45)	0,452
Buona/priva di complicazioni/priva di complicazioni	%, (n)	20.0% (11)	18.2% (10)	
Poco pratica/difficile	%, (n)	3.6% (2)	-	
Impossibile/troppe complicazioni	%, (n)	1.8% (1)	-	
**Il montaggio delle componenti del dispositive, secondo Lei, è stata**				
Priva di problemi	%, (n)	76.4% (42)	96.4% (53)	0,013
Lievi problemi immediatamente risolti	%, (n)	23.6% (13)	3.6% (2)	
Impossibile	%, (n)	-	-	
**Che tipo di problemi ha avuto nel montaggio del dispositive?**				
Nessun problema	%, (n)	78.2% (43)	98.2% (54)	0,988
Problemi nel montaggio dell'apparecchio	%, (n)	12.7% (7)	-	
Problemi nell'accensione dell'apparecchio	%, (n)	1.8% (1)	-	
Problemi nel posizionamento corretto del/la bambino/a	%, (n)	-	-	
Problemi nel posizionamento corretto dell'apparecchio	%, (n)	7.3% (4)	1.8% (1)	
**Come ha tollerato il suo/a bambino/a l'impiego del dispositive**				
Tollerato bene senza alcun problema	%, (n)	76.4% (42)	90.9% (50)	0,106
Dopo alcune iniziali difficoltà lo ha tollerato bene senza	%, (n)	10.9% (6)	5.5% (3)	
Ha pianto o si è agitato inizialmente, ma si è potuto	%, (n)	3.6% (2)	3.6% (2)	
Non ha tollerato l'apparecchio e la nebulizzazione è stata impossibile	%, (n)	9.1% (5)	-	
**La pulizia del dispositive e delle sue componenti è stata:**				
Priva di problemi	%, (n)	85.5% (47)	98.2% (54)	0,939
Lievi problemi immediatamente risolti	%, (n)	12.7% (7)	1.8% (1)	
Alquanto difficile	%, (n)	1.8% (1)	-	
Impossibile	%, (n)	-	-	
**Quando il/la Suo/a bambino/a ha inalato il Ventolin con il dispositive, i suoi sintomi respiratori sono migliorati?**				
Sì: ha potuto respirare liberamente dopo la nebulizzazione	%, (n)	52.7% (29)	70.9% (39)	0,025
Parzialmente ha potuto respirare meglio dopo la	%, (n)	9.1% (5)	14.5% (8)	
No: non ho potuto osservare differenze tra prima e dopo la	%, (n)	10.9% (6)	9.1% (5)	
Non saprei	%, (n)	27.3% (15)	5.5% (3)	
**L'uso del dispositive ha permesso al/la Suo/a Bambino/a di dormire meglio?**				
Si	%, (n)	50.9% (28)	58.2% (32)	0,274
Abbastanza	%, (n)	29.1% (16)	29.1% (16)	
Direi di no	%, (n)	3.6% (2)	3.6% (2)	
Assolutamente no	%, (n)	-	-	
Non saprei, il/la mio/a bambino/a non ha avuto disturbi del sonno	%, (n)	16.4% (9)	9.1% (5)	
**L'uso del dispositive ha migiorato il benessere del/la Suo/a Bambino/a?**				
Si	%, (n)	52.7% (29)	65.5% (36)	0,101
Abbastanza	%, (n)	30.9% (17)	27.3% (15)	
Direi di no	%, (n)	7.3% (4)	3.6% (2)	
Assolutamente no	%, (n)	-	-	
Non saprei	%, (n)	9.1% (5)	3.6% (2)	
**Ritiene che l'uso del dispositive sia stato utile per il/la Suo/a Bambino/a?**				
Si	%, (n)	65.5% (36)	80.0% (44)	0,039
Abbastanza	%, (n)	16.4% (9)	16.4% (9)	
Direi di no	%, (n)	9.1% (5)	1.8% (1)	
Assolutamente no	%, (n)	-	1.8% (1)	
Non saprei	%, (n)	9.1% (5)	-	
**Vorrebbe usare ancora in futuro il dispositive per il/la Suo/a Bambino/a?**				
Si	%, (n)	78.2% (43)	87.3% (48)	0,156
Abbastanza	%, (n)	7.3% (4)	7.3% (4)	
Direi di no	%, (n)	5.5% (3)	3.6% (2)	
Assolutamente no	%, (n)	5.5% (3)	-	
Non saprei	%, (n)	3.6% (2)	1.8% (1)	
**Prima di partecipare a questo studio, aveva utilizzato altri nebulizzatori?**				
No	%, (n)	12.7% (7)	40.0% (22)	0,004
Si	%, (n)	87.3% (48)	60.0% (33)	
**Se SI', ritiene che la praticità e l'efficacia del dispositive sia stato, in confronto con l'impego di altri nebulizzatori:**				
Migliore	%, (n)	83.3% (40/48)	63.6% (21/33)	0,063
Leggermente migliore	%, (n)	8.3% (4/48)	6.1% (2/33)	
Simile	%, (n)	4.2% (2/48)	30.3% (10/33)	
Leggermente peggiore	%, (n)	-	-	
Peggiore	%, (n)	4.2% (2/48)	-	
Non ho utilizzato altri nebulizzatori prima di questo studio	%, (n)	-	-	
**Consiglierebbe l'impiego dell' dispositivel ad altri genitori di bambini con problemi respiratori simili a quelli del/la Suo/a**				
Si	%, (n)	78.2% (43)	89.1% (49)	0,159
Probabilmente si	%, (n)	10.9% (6)	5.5% (3)	
Probabilmente no	%, (n)	7.3% (4)	1.8% (1)	
Assolutamente no	%, (n)	1.8% (1)	-	
Non saprei	%, (n)	1.8% (1)	3.6% (2)	

Figure S1 - The Micro AIR U-100 MESH nebulizer (a) and its functional scheme (b)

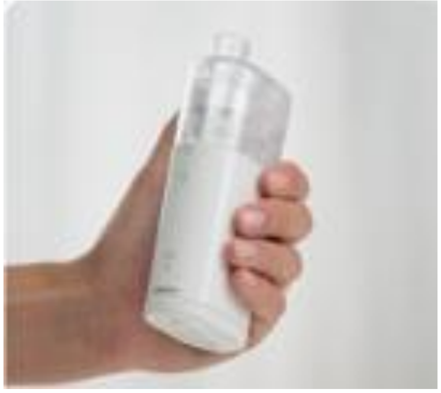

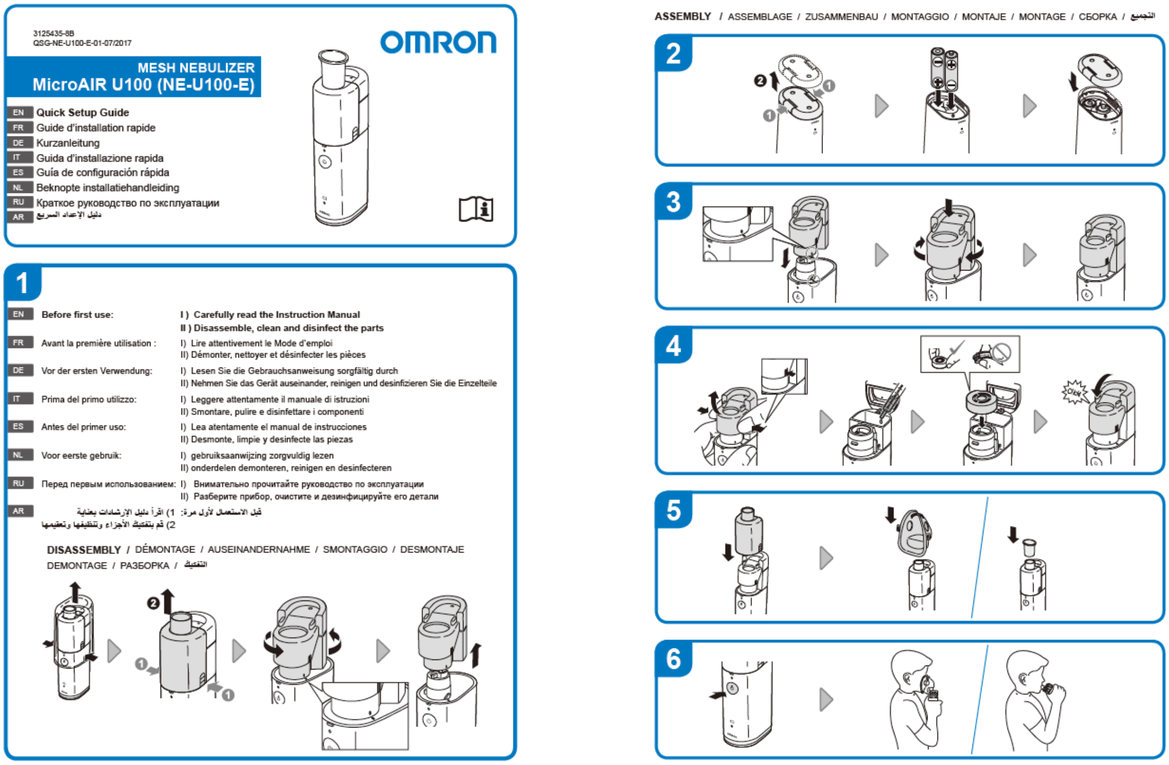

Supplement: Supplementary file 1 [file Data_Sheet_1.pdf]
